# 

**Published:** 1897-02

**Authors:** 


### BOOKS RECEIVED.

Transactions of the American Surgical Association. Volume the fourteenth. Edited by De Forest Willard, A. M., M. D., Ph. D., recorder of the association. Printed for the association. For sale by Wm. J. Dornan, Philadelphia. 1896.

Artificial Anesthesia. A manual of anesthetic agents and their employment in the treatment of disease. By Lawrence Turnbull, M. D., Ph. G., Aural Surgeon to the Jefferson Medical College Hospital, Philadelphia; late Honorary President to the Otological Subsection of the British Medical Association, and of the Section of Laryngology and Otology of the American Medical Association. Fourth edition, revised and enlarged, with illustrations. Philadelphia: P. Blakiston, Son & Co., 1012 Walnut street. 1896.

Over the Hookah. The tales of a talkative doctor. By G. Frank Lydston, M. D., Fellow of the Chicago Academy of Medicine, the Southern Surgical and Gynecological Association, and the American Academy of Social and Political Science; Professor of Criminal Anthropology in the Kent College of Law; Member of the American Medical Association, and the Association of Military Surgeons of the United States; Honorary Fellow of the Texas Medical Association, etc. Illustrated, from the author's designs, by Mr. C. Everett Johnson. Chicago: Fred Klein Co. 1896.

Autoscopy of the Larynx and the Trachea. (Direct examination without mirror.) By Alfred Kirstein, M. D., Berlin. Authorised translation (altered, enlarged and revised by the author,) by Max Thorner, A. M., M. D., Cincinnati, O., Professor of Clinical Laryngology and Otology, Cincinnati College of Medicine and Surgery; Laryngologist and Aurist, Cincinnati Hospital, etc. With twelve illustrations. One volume, crown octavo, pp. xi.—68. Extra cloth, 75 cents net. The F. A. Davis Co., Publishers, 1914 and 1916 Cherry street, Philadelphia; 117 W. Forty-second street, New York; 9 Lakeside Building, Chicago.

A Guide to the Clinical Examination of the Blood for Diagnostic Purposes. By Richard C. Cabot, M. D. With colored plates and engravings. $4.75. New York: William Wood & Co. 1897.

Anomalies and Curiosities of Medicine. Being an encyclopedic collection of rare and extraordinary cases, and of the most striking instances of abnormality in all branches of medicine and surgery, derived from an exhaustive research of medical literature from its origin to the present day, abstracted, classified, annotated and indexed. By George M. Gould, A. M., M. D., and Walter L. Pyle, A. M., M. D. Imperial octavo, 968 pages, with 295 illustrations in the text and twelve half-tone and colored plates. Prices, cloth, $6.00 net; half-morocco, $7.00. Sold only by subscription. Philadelphia: W. B. Saunders, 925 Walnut street. 1897.

The Diseases of Infancy and Childhood. By L. Emmett Holt, A. M., M. D., Professor of Diseases of Children in the New York Polyclinic; Attending Physician to the Babies' Hospital and to the Nursery and Child's Hospital, New York; Consulting Physician to the New York Infant Asylum and to the Hospital for Ruptured and Crippled. Illustrated with nineteen full-page colored photogravure plates and 185 cuts inserted with the text. Sold only by subscription. Prices, cloth, $6.00; sheep, $7.00; half-morocco, $7.50. New York: D. Appleton & Co., Publishers, 72 Fifth avenue.

Report of the Commissioner of Education for the year 1894–95. Volume II., containing Parts II. and III. Washington: Government Printing Office. 1896.

Two Health-seekers in California. By William A. Edwards, M. D., Fellow of the College of Physicians of Philadelphia, etc., etc., and Beatrice Harraden, author of Ships that Pass in the Night, etc. Philadelphia: J. B. Lippincott & Co. 1897.

